# Mid-term safe and effective profile of the Magmaris scaffold in percutaneous coronary intervention: a prospective, single-center study

**DOI:** 10.3389/fcvm.2023.1194933

**Published:** 2023-05-26

**Authors:** Binh Quang Truong, Hoa Tran, Vinh Xuan Nguyen, Chinh Duc Nguyen, Khang Duong Nguyen, Vu Hoang Vu

**Affiliations:** ^1^ Medicine Faculty, University of Medicine and Pharmacy at Ho Chi Minh City, Ho Chi Minh City, Vietnam; ^2^ Interventional Cardiology Department, University Medical Center Ho Chi Minh Center, Ho Chi Minh City, Vietnam

**Keywords:** Magmaris sirolimus-eluting bioresorbable scaffold, intravascular ultrasound, percutaneous coronary intervention, prospective and interventional study, scaffold thrombosis

## Abstract

**Introduction:**

Significant advances have been made in the diagnosis and treatment of coronary artery disease over the years. New generations of scaffolds containing novel material and eluting drug have produced one of the most significant advancements in coronary intervention. The newest generation would be Magmaris with a magnesium frame and a sirolimus cover.

**Methods:**

From July 2018 to August 2020, 58 patients treated with Magmaris at the University Medical Center Ho Chi Minh City were enrolled in this study.

**Results:**

A total of 60 lesions were stented, 60.3% of which were left anterior descending (LAD) lesions. There was no in-hospital event. Within 1 year after discharge, we noted one myocardial infarction event that required target-lesion revascularization, one stroke event, one non-target-lesion revascularization patient, two target-vessel revascularization patients, and one in-stent thrombosis. Among them, one myocardial infarction occurrence, one non-target-lesion revascularization, and one in-stent thrombosis event were recorded within the first 30 days after discharge.

**Conclusion:**

In conclusion, the Magmaris scaffold is a safe and effective option for structural procedures performed with imaging device support, particularly intravascular ultrasound.

## 1. Introduction

Cardiovascular diseases remain a leading cause of morbidity and mortality in general population and in non-transmitted diseases. The management of this disease through percutaneous coronary angiography and intervention has witnessed significant advancements from technical issues in coronary balloon angioplasty to bare metal stent and then newer generations of drug-eluting scaffolds. Recently, in the efforts to improve drug-eluting scaffold disadvantages, which would be loss of physiological functions and the permanence of steel material within the coronary artery, bioabsorbable stents have been introduced. The self-sustaining scaffold has since also been expected to be the fourth revolution in percutaneous coronary intervention. In actuality, the first generation of the self-sustaining scaffold, called Absorb, with a design of a tubular scaffold frame made of poly-L-lactide acid and zigzag scaffold eyes, has resulted in inferior angiographic and clinical outcomes at 3 years (1), and also an increase in the target failure and stent thrombosis rate between 1 and 3 years, and combined over 3 years, when compared with the everolimus-coated scaffold (2).

The Magmaris is the next representative of the bioabsorbable scaffold and is seen as a generation that outperforms certain improvements, from the magnesium support frame to the sirolimus mantle. Along with well-anticipated features, positive results were noted in early studies (3). These results also took Magmaris to new heights following the failures of the previous generation, Absorb (4). The Magmaris scaffold and its new structure, new design and thorough lesion preparation instruction, have overcome the disadvantages of the bioabsorbable first-generation stent, while preserving the advantages of a conventional drug-eluting one. In a developing country such as Vietnam, the collection of data on the safety and effectiveness of Magmaris is of great significance in the treatment of cardiovascular disorders, helping to develop a more appropriate monitoring and intervention plan.

## 2. Methods

### 2.1. Study device

The Magmaris system contains of a proprietary magnesium alloy in the scaffold, with two tantalum markers at each end. The scaffold was coated with resorbable poly-L-lactide eluting a limus drug with a dose of 1.4 μg/mm^2^ and a strut thickness of 150 μm. The technical data of the delivery system are presented in Table 1.

**Table 1:** Technical data of the Magmaris delivery system.

Catheter type	Rapid exchange
**Recommended guide catheter**	6F
**Crossing profile**	1.5 mm
**Guide wire catheter**	0.014 in.
**Usable catheter length**	140 cm
**Balloon material**	Semi-crystalline polymer
**Coating (distal shaft)**	Dual coated
**Marker bands**	Two swaged platinum–iridium markers
**Proximal shaft diameter**	2.0F
**Distal shaft diameter**	2.9F
**Nominal pressure**	10 atm
**Rated burst pressure**	16 atm

The stent is also available in two diameters, 3.0 and 3.5 mm, and three length sizes, 15, 20, and 25 mm.

### 2.2. Study design

**Figure 1:**
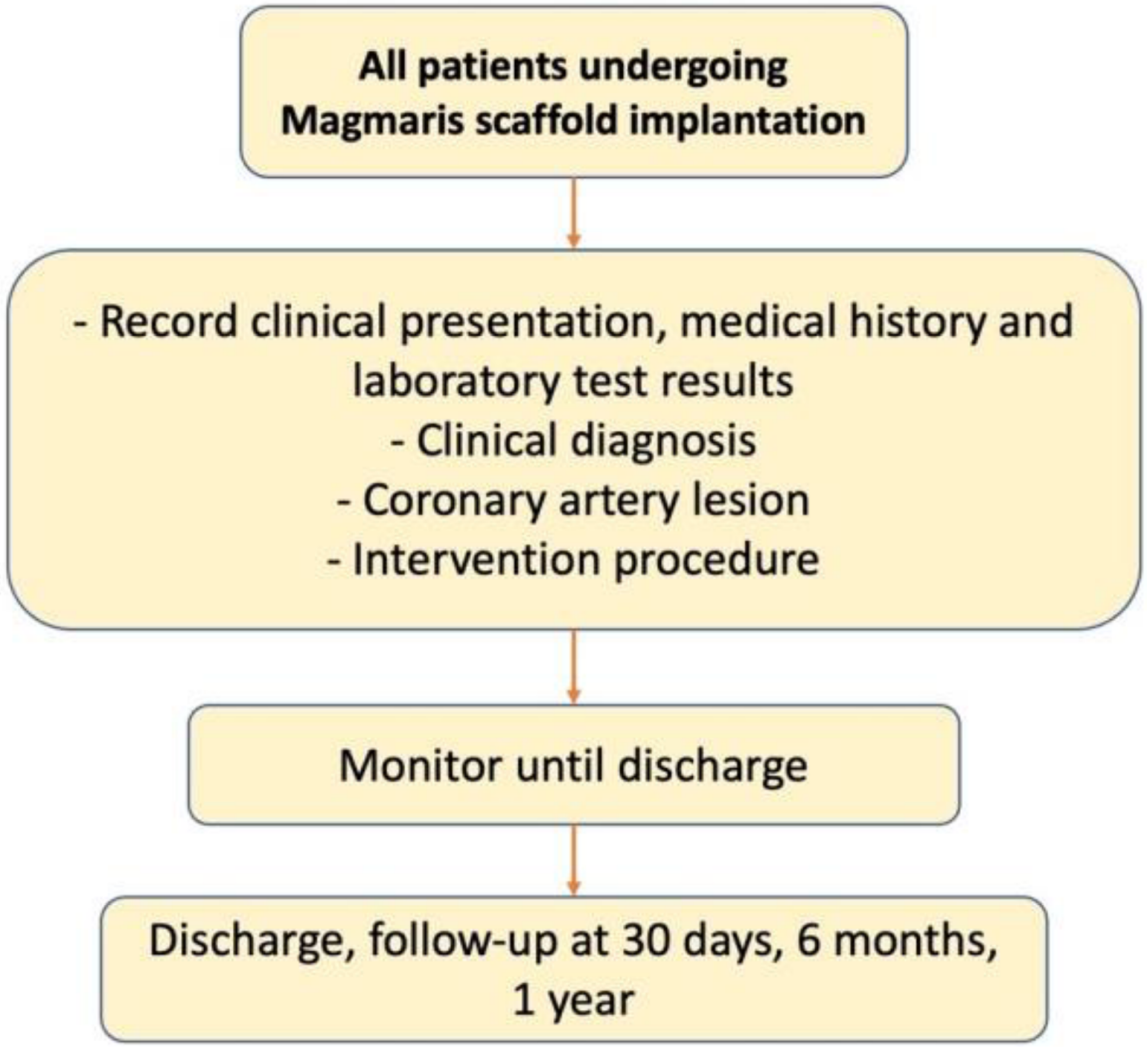
Study's flow chart.

We conducted a single-center cohort study on percutaneous coronary intervention patients with magnesium scaffold, sirolimus-eluting bioabsorbable Magmaris at the University Medical Center Ho Chi Minh City, from July 2018 to August 2020 (Figure 1). The inclusion criteria included hospitalized patients with coronary artery disease with a maximum reference vessel diameter of 3.0–3.75 mm [intravascular ultrasound (IVUS) measurement]. The main exclusion criteria included cardiogenic shock, left coronary artery disease or severe calcific lesion, and patients with comorbidities that prolong survival was estimated at < 1 year.

We collected patient information from past medical history, presentation, and laboratory results as well as data on the intervention procedure including coronary lesions and the process of percutaneous coronary intervention. The procedure for percutaneous coronary intervention with Magmaris scaffold was closely followed, which consists of four steps: patient selection, proper sizing, pre-dilatation, and post-dilatation. All patients after percutaneous coronary intervention were observed until discharge and followed up at 30 days, 6 months, and 1 year after discharge. The primary goal of the study was to evaluate the effectiveness and safety of the Magmaris during hospitalization and after 6 months and 1 year of discharge. Efficacy was assessed through the likelihood of major cardiac events (death, myocardial infarction, and stroke) during monitoring, and safety was assessed by the rate of procedure-related complications during hospitalization and the rate of target-vessel revascularization, target-lesion revascularization, and in-stent thrombosis during observation.

Prior to stent implantation, intravascular ultrasound was carried out. Prior to stenting, intravascular imaging can evaluate plaque composition and distribution (calcification, lipid-rich plaque), identify the need for more aggressive (rotational atherectomy, cutting, or scoring balloons to induce calcium fractures) or less aggressive (direct stenting to avoid lipid embolization) lesion preparation, and facilitate stent size selection (diameter, length). Imaging was performed using a motorized pullback device, with continuous control over the image quality throughout the acquisition process. Occasionally, IVUS requires a manual pullback to confirm focal and specific findings detected during the automatic pullback. Imaging catheters with a low profile and an exposed lumen must be purged to remove air and ensure optimal image quality. The imaging run began at least 20 mm distal to the lesion and ended at the left main or right coronary artery (RCA) ostium to include the longest vessel segment possible. If the imaging catheter was unable to cross the lesion prior to stenting, balloon pre-dilatation may be used to facilitate image acquisition.

The diagnosis of myocardial infarction was based on the fourth definition of myocardial infarction (5), in which myocardial infarction is defined as the presence of acute myocardial injury detected by abnormal cardiac biomarkers in the presence of evidence of acute myocardial ischemia.

The criteria for scaffold thrombosis were adapted from the Academic Research Consortium-2 document as presented in Table 2 (6).

**Table 2:** Definition and timing of stent/scaffold thrombosis (6).

Classification	Criteria
Definite stent/ scaffold thrombosis	Angiographic confirmation of stent/scaffold thrombosis
	The presence of a thrombus that originates in the stent/scaffold or in the segment 5 mm proximal or distal to the stent/scaffold or in a side branch originating from the stented/scaffolded segment and the presence of at least one of the following criteria:
	Acute onset of ischemic symptoms at rest
	New electrocardiographic changes suggestive of acute ischemia
	Typical rise and fall in cardiac biomarkers (refer to definition of spontaneous myocardial infarction)
	Or
	Pathological confirmation of stent/scaffold thrombosis
	Evidence of recent thrombus within the stent/scaffold determined at autopsy
	Examination of tissue retrieved following thrombectomy (visual/histology)
Probable stent/ scaffold thrombosis	Regardless of the time after the index procedure, any myocardial infarction that is related to documented acute ischemia in the territory of the implanted stent/scaffold without angiographic confirmation of stent/scaffold thrombosis and in the absence of any other obvious cause.
Silent stent/ scaffold occlusion	The incidental angiographic documentation of stent occlusion in the absence of clinical signs or symptoms is not considered stent thrombosis.
Timing of ST (duration after stent implantation)	
Acute	0–24 h
Subacute	>24 h–30 days
Late	>30 days–1 year
Very late	>1 year

The criteria of angiographic success were defined as final minimum stenosis diameter reduction to < 10%, and a procedure success was defined as angiographic success without in-hospital major clinical complications (e.g., death, myocardial infarction, and emergency coronary artery bypass surgery) (7).

Table 3 shows the criteria used in this study to determine the optimal result of scaffold implantation.

**Table 3:** Optimal stent result criteria (8).

• A relative stent expansion of >90% (MSA divided by distal reference lumen area) should be obtained in routine clinical practice. • An MSA of >5.5 mm^2^ by IVUS should be achieved in non-left main lesions. • The clinical relevance of acute malapposition is uncertain. Nonetheless, extensive malapposition after stent implantation should be avoided and corrected, if anatomically feasible. Early strut coverage may be promoted by full apposition. • Acute malapposition of <0.4 mm with longitudinal extension <1 mm or malapposition should not be corrected as spontaneous neointimal integration is anticipated. This cut-off requires prospective validation. • Late acquired malapposition represents an established cause of late and very late stent thrombosis. • Tissue prolapse in ACS as compared with stable CAD is adversely related to outcomes, likely because of differences in the composition of the protruding tissue. • Large dissections detected by IVUS are independent predictors of MACE. Presence of residual plaque burden, extensive lateral (>60°), and longitudinal extension (>2 mm), involvement of deeper layers (medial or adventitia) and localization distal to the stent increase the risk for adverse events. • Stent edge hematoma may be detected by IVUS in case of angiographic appearance of a residual stent edge stenosis.

Data in the study were imported into the Excel 2010 software, with R being used to access and analyze data. Qualitative variables are shown through the frequency and proportional distribution table. Quantitative variables with standard distribution are represented by mean ± standard deviation, and no standard distribution is represented by medians.

This research has been approved by the Ethics Council for research on biomedicine of the University of Medicine and Pharmacy at Ho Chi Minh City, 53/GCN-HDĐ. Patients who had taken part in the study had signed a consent form to their participation.

## 3. Results

### 3.1. Patients characteristics

**Figure 2:**
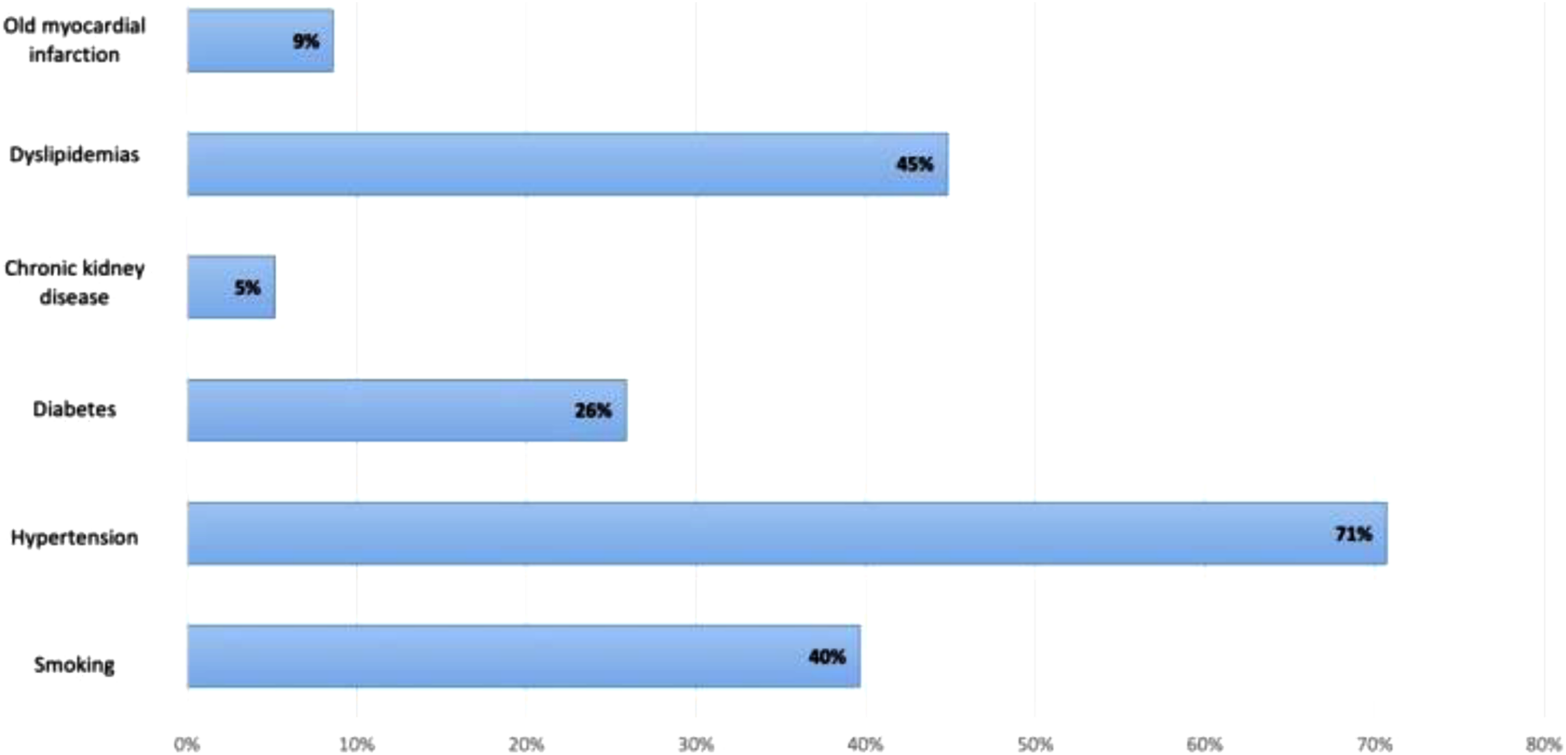
Coexisting disorders prevalence.

Our study enrolled 58 patients with 60 lesions intervened with Magmaris. The average age of the patients in the study was 59.4 ± 10.4 years (37; 86). Majority of the study population were male with a total number of 41 patients (71%). Coexisting disorders are depicted in Figure 2, with hypertension accounting for the highest proportion (71%).

Most patients were hospitalized with non-ST-segment elevation coronary syndrome (67%); only 6 patients (10%) had ST-segment elevation myocardial infarction, and 13 patients (22.4%) had chronic coronary syndrome.

Some of the laboratory and echocardiography features are shown in Table 4.

**Table 4:** Basic laboratory test results.

*N* = 58	Minimum	Maximum	Mean ± SD
Cholesterol (mg/dl)	73.5	284.6	178.32 ± 44.97
LDL (mg/dl)	40	200.4	113.55 ± 32.41
Creatinine (mg/dl)	0.6	1.75	0.96 ± 0.19
NT-proBNP	9	4,704	370.53 ± 764.89
hs-troponin T (ng/L)	3.9	1,682	101.94 ± 264.26
CK-MB (ng/L)	5	135	21.78 ± 22.07
Glucose (mg/dl)	83	323	138.17 ± 58.97
LVEF (%)	42	80	63.62 ± 8.87

### 3.2. Intervention characteristics

We performed the intervention on 60 lesions of 58 patients; 1 of them had three Magmaris implanted. The left anterior descending branch was the most commonly affected (38 lesions, 60.3%). Among the cases with complex lesions, there were 3 complete total occlusion (4.8%) and 3 ostial stenosis lesions (4.8%). The features on coronary angiography and revascularization are shown in Tables 5 and 6.

**Table 5:** Lesions characteristics.

	*N* = 58
Lesion location	
LAD	38/60 (63.3%)
LCx	10/60 (16.7%)
RCA	14/60 (23.3%)
Number of affected coronary artery	
1	22/58 (37.9%)
2	23/58 (39.7%)
3	13/58 (22.4%)
ACC/AHA lesion classification	
A	2/60 (3.3%)
B1	31/60 (51.7%)
B2	16/60 (26.7%)
C	11/60 (18.3%)
Mixed lesion	54/60 (90%)
Chronic total occlusion	0/60 (0%)
Bifurcation lesion	0/60 (0%)

**Table 6:** Coronary intervention characteristics.

	*N* = 58
Number of patients:	58
Mean heparin dose (UI)	6333
Pre-dilatation balloon: Balloon diameter (mm) Balloon pressure (atm)	2.85 ± 0.35 13.93 ± 2.23
Number of scaffolds:	60
Scaffolds size (mm)	
3.0 × 15	5
3.0 × 20	9
3.0 × 25	11
3.5 × 15	6
3.5 × 20	15
3.5 × 25	14
Scaffold pressure (atm)	13.62 ± 2.42
Post-dilatation balloon: Balloon diameter (mm) Balloon pressure (atm)	3.53 ± 0.29 18.03 ± 1.97
Intervention success:	100%
IVUS	98.3%

**Figure 3:**
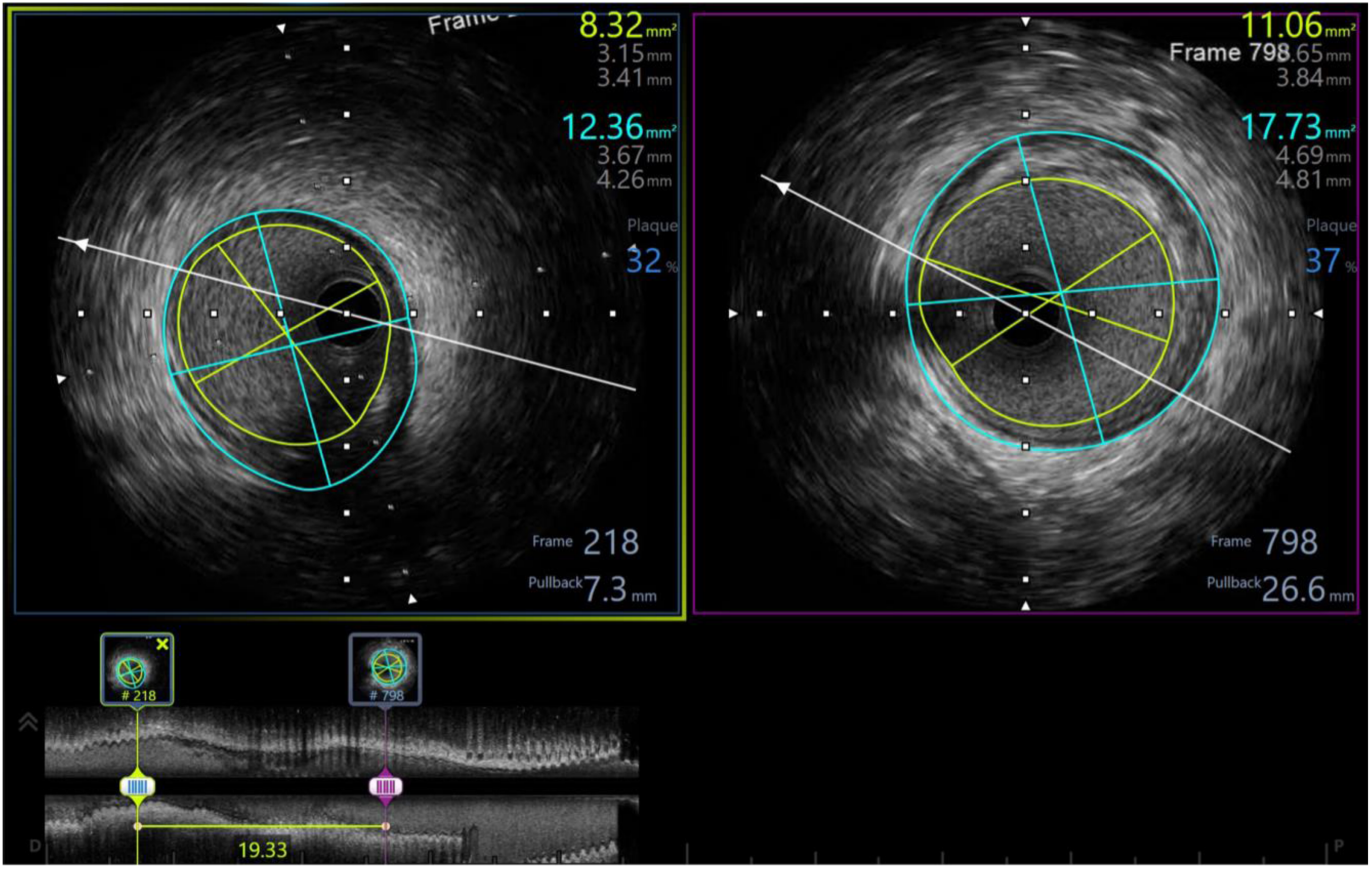
Pre-procedure IVUS showed distal reference: lumen diameter 3.15  ×  3.41 mm, vessel diameter: 3.67  ×  4.26, plaque burden: 32%; proximal reference: lumen diameter 3.65  ×  3.84 mm, vessel diameter: 4.69  ×  4.81, plaque burden: 37%; lesion length: 19 mm.

**Figure 4:**
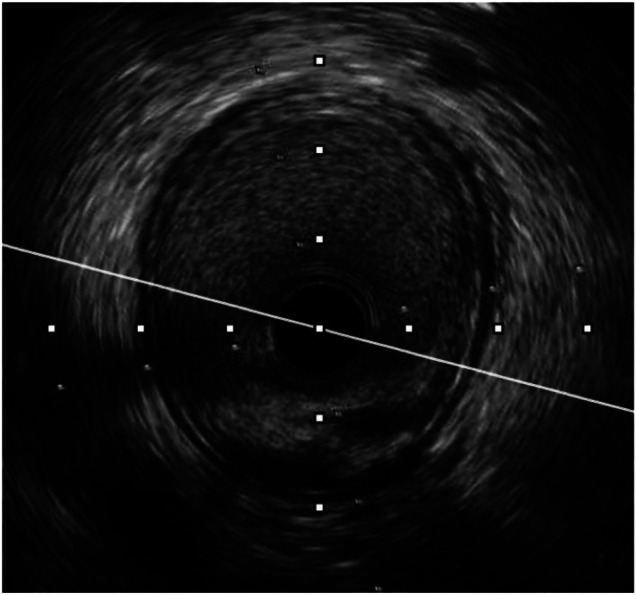
Pre-procedure IVUS of the same patient (from Figure 3) showed fibrotic lesion with plaque rupture.

**Figure 5:**
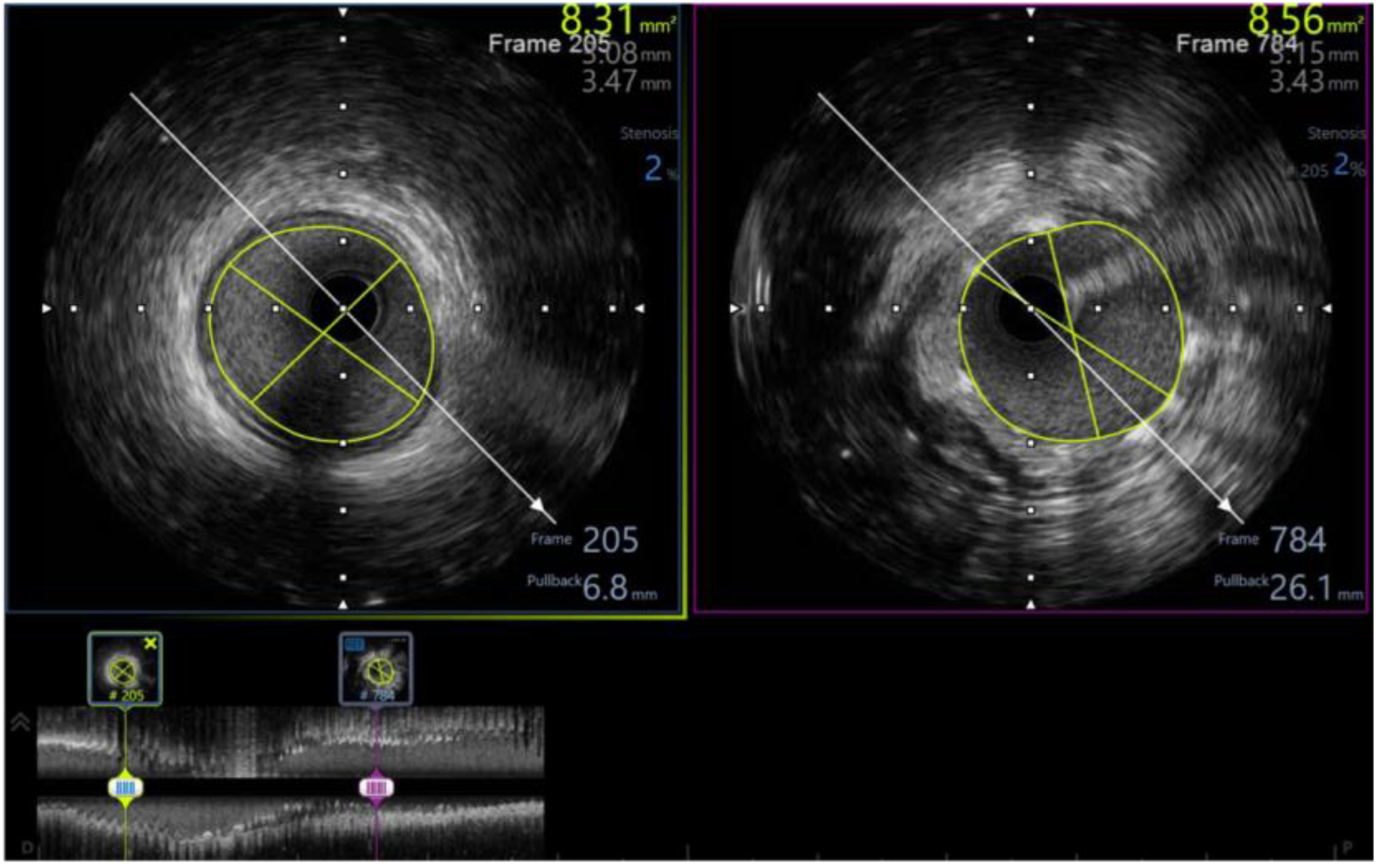
Post-procedure IVUS of the same patient (from Figures 3, 4) showed minimum stent area of 8.56 mm^2^ (# 102% distal reference lumen area) with well apposition, no tissue protrusion, and no edge dissection.

In our study, 98.3% of patients received intravascular ultrasound prior to implantation to evaluate the lesion characteristics and to select the appropriate scaffold size, as well as an intravascular ultrasound after the intervention to evaluate the effectiveness and success rate of the procedure. All of the patients received post-intervention balloon dilation. The efforts to follow the same process have helped the procedure to achieve a successful intervention rate of 100% (Table 6). Figures 3–5 displayed IVUS imaging of a patient who underwent Magmaris implantation using IVUS.

### 3.3. Anti-platelet usage

After a year of prescribing a dual anti-platelet therapy to all of our patients, we transitioned to a single anti-platelet therapy. Aspirin was administered to each patient (100%). Regarding P2Y12 inhibitor, majority of patients were prescribed with Clopidogrel (68.9%), followed by Ticagrelor (31.1%).

### 3.4. In-hospital and follow-up outcomes

**Figure 6:**
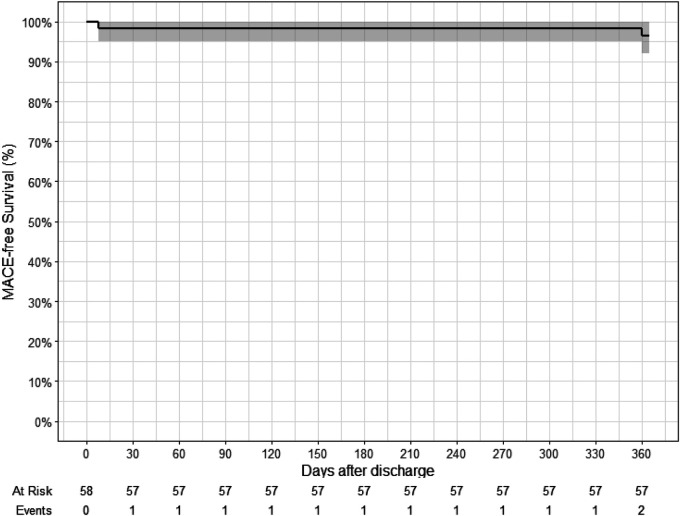
Kaplan–Meier curve for major adverse cardiac events (death, myocardial infarction, and stroke): 1 patient had myocardial infarction 8 days after discharge; 1 patient had stroke 360 days after discharge.

We did not document any in-hospital events, including major cardiovascular events (such as death, myocardial infarction, and stroke). The outcomes and events in all patients within the hospital and 30 days and 1 year after discharge are shown in Table 7 and Figure 6.

**Table 7:** In-hospital, 30 days, and 1 year after discharge outcomes.

	In-hospital (*N* = 58)	30 days post-discharge (*N* = 58)	1 year post-discharge (*N* = 58)
Death	0 (0%)	0 (0%)	0 (0%)
Myocardial infarction	0 (0%)	1 (1.7%)	1 (1.7%)
Stroke	0 (0%)	0 (0%)	1 (1.7%)
Target-lesion revascularization	0 (0%)	0 (0%)	1 (1.7%)
Non-target-lesion revascularization	0 (0%)	1 (1.7%)	1 (1.7%)
Target-vessel revascularization	0 (0%)	0 (1.7%)	2 (3.4%)
In-stent thrombosis	0 (0%)	0 (0%)	1 (1.7%)

All events are described in the following manner: 1 patient implanted with Magmaris in the proximal LAD had NSTEMI 8 days after discharge, and a drug-eluting stent (DES) was inserted in the OM1 segment. Within 30 days, this occurrence was classified as myocardial infarction and non-target-lesion revascularization. Another patient had 3 Magmaris scaffolds inserted (2 at the proximal LAD and 1 at the middle LAD artery). He was readmitted after 37 days with unstable angina. IVUS performed at the time detected thrombosis within the scaffold. At the same time, he was diagnosed with atrial fibrillation; therefore he was treated with anticoagulants. The same patient suffered from unstable angina 351 days after the initial hospitalization and was diagnosed with middle LAD aneurysm with 70% pre-stent stenosis; thus, one DES was implanted at the middle LAD. These events were classified as in-stent thrombosis, target-lesion revascularization, and target-vessel revascularization within 1 year. We also documented one case in which a Magmaris scaffold was implanted in the distal RCA artery; the patient returned after 206 days with an episode of unstable angina. Afterwards, a DES was implanted in the patient's middle RCA. Hence, he was accounted for an occurrence involving target-vessel revascularization within a year. In addition, we observed one ischemic stroke within 1 year of discharge.

## 4. Discussion

Drug-eluting stents are now the gold standard in percutaneous coronary revascularization. There were some disadvantages in relation to the stent that existed permanently in the vessel. This study was done to evaluate the efficacy and safety of the bioabsorbable scaffolds, which were developed to overcome the limitations of the permanent ones. Magmaris has been demonstrated to be a safe and effective alternative comparable with the findings of global studies.

Magmaris was not really the first attempt by coronary interventionists to invent a new-generation bioabsorbable scaffold. The magnesium-framed absorbable stent AMS 1 (BIOTRONIK, Berlin, Germany) was the initial bare version with a strut thickness of 165 μm. It was evaluated in the PROGRESS AMS FIM study on 63 patients and resulted in a disappointing target-lesion revascularization rate of 45% after 1 year despite the absence of in-stent thrombosis event, thus sparking the need to redesign this device (9). The next generation AMS 2 (DREAMS 1) used a refined WE43 alloy (93% magnesium and 7% rare elements) with a slower bio-absorption time (9–12 months), higher radial intensity, 125 μm struts with a rectangular shape, and 3-month-release paclitaxel coating. It was tested in the BIOSOLVE I study (10), noting a late lumen loss in coronary angiography of 0.52 ± 0.49 mm at 1 year; while at 3 years the rate of target-vessel revascularization reached 6.6%, there were no cases of cardiac death, myocardial infarction in the target vessel, or early thromboembolism in the scaffold.

In an attempt to further improve the scaffold quality, a new version (second generation DREAMS, marketed as Magmaris) has been in development recently, which was made of alloy and similar design but with a strut thickness of 150 μm. This scaffold overlays sirolimus instead of paclitaxel, which has tantalum radionuclide markers on both ends and a modified, electro-polished strut cross-section. There was no increase in recoil after 1 hour (11), and 95% of the magnesium was reabsorbed at 12 months (12) as opposed to 3 years of the Absorb (13), which means it reduces the risk of neoatherosclerosis more rapidly. The Magmaris was evaluated in the international multicenter FIM BIOSOLVE II trial (*n* = 123) (3). Late lumen loss was 0.27 ± 0.37 mm after 6 months (primary endpoint) and 0.39 ± 0.27 mm at 12 months track. The major outcome of cardiac death, target-vessel myocardial infarction, or early in-stent thrombus, and coronary artery bypass grafting occurs in four patients (3%), including one cardiac death, one destination-vessel infarction, and two clinically based revascularization. The long-term results of Magmaris BRS were recently published, including a 24-month composite outcome data from BIOSOLVE II (*n* = 123) and BIOSOLVE III (*n* = 61) trial (14), which demonstrated the target-lesion failure rate (defined as cardiac death, target-vessel myocardial infarction, and revascularization) of 3.3% at 12 months and 5.6% over 2 years. Our study reported no fatalities, and the target-lesion failure rate was 3.4% at 12 months. These figures, similar to other studies worldwide, once again confirmed the effectiveness of Magmaris scaffold, especially in the Asian population group. In 2020, the BIOSOLVE IV multi-center study (15) that collected 1,075 patients with 1,121 lesions followed up for up to 5 years with the main result of target-lesion failure rate at 12 months (4.3%) confirmed the efficiency and safety of the Magmaris in a large population with a high success rate in terms of equipment and procedures as well as a safety record of up to 12 months on a low risk population. The primary outcomes of cardiac deaths, target-vessel myocardial infarction, early in-stent thrombosis, or coronary artery bypass graft were seen in four patients (3%).

One point to note when comparing our study with preexisting data is the characteristics of the study population. Our population had higher rates of hypertension (71% compared with 67.3%), higher rates of diabetes (26% compared with 21.2%), and 77% of the population were admitted to hospital with acute MI compared with 19.2% in the BIOSOLVE IV study (16). The rate of lesion classification B2/C according to AHA/ACC in our population was three times higher than that of BIOSOLVE IV (45% vs. 15.2%), and the length of lesion was also longer (18.4 ± 3.3 vs. 14.9 ± 4.2 mm). A significant difference in our study from BIOSOLVE II, III, and IV was the proportion of patients receiving intravascular ultrasound before and after intervention: 98.3% compared with only when imaging on coronary angiography is unclear. The exact ratio of the number of intravascular ultrasound was also unreported (3, 14). Futhermore, the importance of using intravascular imaging to reduce the risk of stent thrombosis with bioresorbable vascular scaffolds has been proposed (17). This suggests that intravascular ultrasonography is an effective tool and should be considered for use in the majority of patients to prepare the lesion and select the appropriate stent size. Besides, optical coherence imaging, which has a higher resolution than intravascular ultrasound, may be a viable option for enhancing the detection of dissection and malposition (18). In addition, the application of the Magmaris in practice may be extended to be indicated even in the higher risk populations, as well as in ST segment elevation MI due to early positive results in both safety and efficacy outcomes.

The success of the Magmaris needs to be compared with the previous generation of self-targeting scaffolds, Absorb, in order to see the differences and why these differences are clinically significant. The Absorb scaffold is composed of entirely resorbable poly-L-lactide acid and poly D, L-lactide-based everolimus coating (19). However, polymer-based stents continue to be problematic due to their inadequate mechanical strength and easy elastic shrinkage, extended degradation time (approximately 3 years compared with 1 year for the Magmaris), and poor vascular compliance (20). Polymer stents have lesser resilience and mechanical strength than metallic stents; hence, polymer stents need to be larger to achieve the same mechanical performance. As an effort to prove efficacy, the Absorb scaffold was evaluated alongside a cobalt–chromium everolimus-eluting stent, with poor results. The 3-year rates of target-lesion failure were higher [11.7% vs. 8.1%; risk ratio (RR), 1.38; 95% confidence interval (CI), 1.10–1.73; *P* = 0.006], driven primarily by higher target-vessel myocardial infarction (7.8% vs. 4.2%; RR, 1.72; 95% CI, 1.26–2.35; *P* = 0.0006) and ischemia-driven target-lesion revascularization (6.6% vs. 4.4%; RR, 1.44; 95% CI, 1.05–1.98; *P* = 0.02) (2). So, the difficulty with Magmaris was what differences it could make to produce beneficial effects. With the Magmaris scaffold, the 4P technical procedure is strictly followed. First, in patient selection, this step is critical for procedural success. It is currently indicated for the *de novo* lesion with reference blood vessel size and length of the lesion approximately the size of the existing Magmaris. The next step is proper sizing. The basic principle is that the image on the coronary angiography mostly underestimates the coronary artery size (21). With the existing Magmaris scaffold diameter of 3.0 mm and 3.5 mm, the choice of a suitable size is very important, because it would be inappropriate if we implant it into the vessel with < 2.7 mm or > 3.7 mm diameter. The use of intravascular ultrasound at a rate of 98.3% in our study made the size selection the most accurate. The last two steps are the use of pre- and post-dilatation balloon, which help optimize vascular implantation. With the help of 100% of patients who received balloon dilatation before and after scaffold implantation as well as 98.3% of patients receiving intravascular ultrasound to evaluate their post-intervention effectiveness, Magmaris was considered a major success. These steps are completely different from the Absorb, when the procedure was initially patient-unlimited, the ratio of intravascular ultrasound in size selection as well as post-intervention assessment was low (23.9%), and balloon dilatation was optional (66.2%) (2). Consequently, despite having the same strut thickness of 150 μm, Magmaris is superior to Absorb in two aspects: design improvements with 1-year fully resorbable material; magnesium-based alloy as opposed to a polymer-based scaffold; and technical improvements with the 4P principle under the guidance of intravascular ultrasound. These distinctions contributed significantly to the reduced rate of target-lesion revascularization and scaffold thrombosis in comparison with Absorb. The fact that 95% of the magnesium in Magmaris was reabsorbed after 12 months (12) is an advantage over Absorb, which required dual therapy for up to 3 years (13).

Our study has certain limitations. First, this is a single-center study, so the representativeness of the population will not be optimal. However, the study was conducted in one of the largest coronary intervention centers in Vietnam, so the study remains valuable in practice and serves as a stepping stone for larger studies. Secondly, the maximum period of observation for 1 year is relatively short. Therefore, even if positive results were recorded after 1 year, the safety and effectiveness should be assessed for a longer time. Finally, despite already having up to 45% of type B2/C lesions, the selection bias introduced by the inclusion/exclusion criteria of this study might prompt interventionists to choose less complex lesions for Magmaris implantation.

## 5. Conclusion

The results of the Magmaris scaffold in our study emphasized superior safety over the previous generation of bioabsorbable scaffold. In order to achieve a procedural and clinical success, the procedure for placing a Magmaris should adhere to the principle of the 4P: patient selection, proper sizing, pre-dilatation, and post-dilatation balloon. Intravascular ultrasound can be a useful and important tool. Further randomized trials are required to ascertain whether angio-guided Magmaris implantation and IVUS-guided Magmaris implantation have distinct clinical outcomes in clinical practice.

## Data Availability

The raw data supporting the conclusions of this article will be made available by the authors, without undue reservation.
